# Oral Malignant Melanoma Achieving Long-Term Survival With Nivolumab Monotherapy Following Surgical Treatment: A Report of a Rare Case

**DOI:** 10.7759/cureus.98037

**Published:** 2025-11-28

**Authors:** Shihomi Kobayashi, Keita Todoroki, Katsuhisa Matsuo, Yushi Abe, Jingo Kusukawa

**Affiliations:** 1 Dental and Oral Medical Center, Kurume University School of Medicine, Fukuoka, JPN; 2 Department of Dentistry and Oral Surgery, Takagi Hospital, Fukuoka, JPN

**Keywords:** maxillary gingiva, melanocytes, nivolumab ipilimimab, oral malignant melanoma, tumor

## Abstract

Oral malignant melanoma (OMM) is a rare and aggressive tumor with a poor prognosis, often presenting with early cervical lymph nodes or distant metastases. We report a case of a 69-year-old woman with primary OMM of the left maxillary gingiva, initially accompanied by indeterminate pulmonary nodules. Following partial maxillectomy, she received nivolumab monotherapy for an enlarged pulmonary nodule and para-aortic lymph nodes. After 11 cycles, a partial response (PR) was achieved in the lung metastasis according to the Response Evaluation Criteria in Solid Tumors (RECIST, version 1.1). However, new metastases later developed in the gallbladder, brain, right frontal scalp, adrenal glands, tongue, and colon. Whole-brain radiation therapy was administered as best supportive care for brain metastases. At the patient’s request, nivolumab monotherapy was continued during the radiation treatment. Notably, regression of the brain metastases was observed after whole-brain radiotherapy while continuing nivolumab, suggesting a potential local synergistic effect. Nivolumab was administered for a total of 21 cycles, and the patient achieved 20 months of survival, which exceeded the previously reported median survival for OMM. This case highlights the aggressive clinical behavior of OMM, its capacity to metastasize to multiple organs, and the potential benefits of immune checkpoint inhibitors (ICIs) and radiation therapy. Further accumulation of cases is needed to define optimal treatment strategies for this rare malignancy.

## Introduction

Oral malignant melanoma (OMM) is a rare tumor originating from melanocytes in the oral mucosa, accounting for approximately 0.2-8% of all malignant melanomas (MMs) [1]. OMM is characterized by its aggressive behavior, with early cervical lymph node metastasis (50%) and distant metastases reported in the lungs (38-53%), bones (36%), brain (20%), and liver (20%) [1]. The median survival period of OMM is approximately six to nine months [2-4], and it is classified as a highly malignant tumor.

Although the treatment of MM generally follows the guidelines provided by the Japanese Skin Cancer Society [5,6], there is no established consensus regarding pharmacotherapy or radiotherapy for OMM. Surgical resection is often chosen for resectable OMM [7,8]. However, in cases with unresectable tumors or distant metastases, multidisciplinary treatment including surgery, pharmacotherapy, and radiotherapy may be applied depending on the patient’s general condition [8].

In recent years, treatment options for MM have expanded significantly, with immune checkpoint inhibitors (ICIs) (e.g., nivolumab, ipilimumab) and targeted therapies (such as BRAF inhibitors, MEK inhibitors, and C-KIT inhibitors) now being utilized as adjuvant treatments [9,10]. However, the occurrence of BRAF mutations in mucosal melanomas, including OMM, has been reported to be less than 1%, thereby limiting treatment options compared with those available for cutaneous MM.

In this report, we present a case of primary OMM of the left maxillary gingiva, initially accompanied by indeterminate pulmonary nodules. The patient underwent partial maxillectomy followed by nivolumab monotherapy, resulting in a survival duration of 20 months after the initial diagnosis. To the best of our knowledge, no cases of long-term survival have been reported with nivolumab monotherapy following surgical treatment for OMM. We also discuss the relevant literature on this topic.

## Case presentation

In December 2022, a 69-year-old woman with a history of hypertension, hyperlipidemia, and a 28-year history of smoking (10 cigarettes per day since age 27) presented with a pigmented lesion of the left maxillary gingiva. Clinical examination revealed an irregular dark brown macule, measuring 20 × 15 mm, centered on the gingiva around the upper left second premolar and first molar. The lesion had poorly defined margins, and scattered light brown pigment deposits were present around the primary lesion discontinuously. No cervical lymphadenopathy was palpable. Panoramic and dental X-rays showed no evidence of bony destruction in the area of the primary site. At the initial visit, the lesion appeared clinically uncertain, making it difficult to distinguish between benign (e.g., pigmented nevus or oral melanosis) and malignant characteristics. We decided to implement a close follow-up strategy and planned for imaging and biopsy if any changes were observed. 

**Figure 1:**
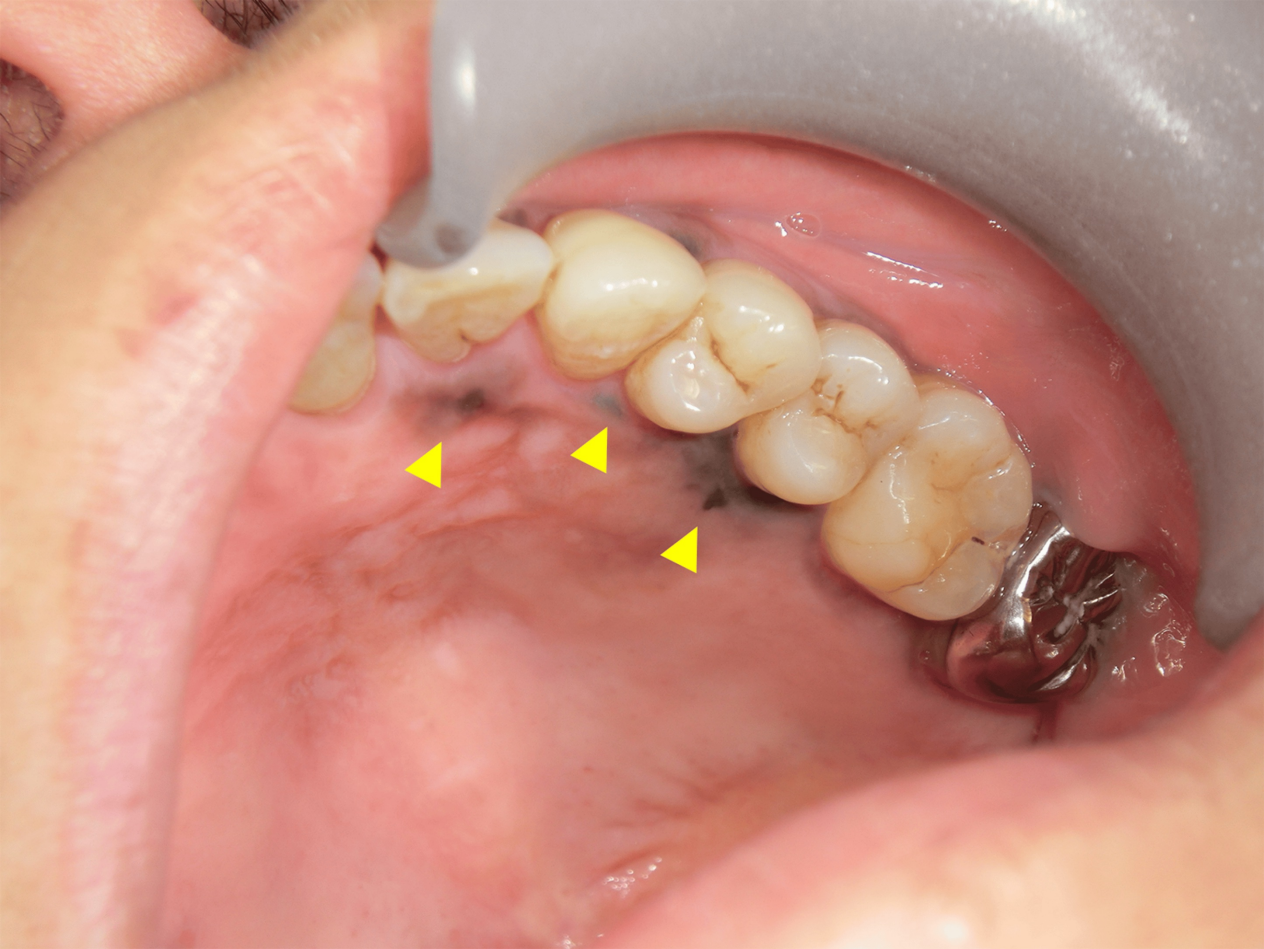
Intraoral view showing an irregular dark brown macule (20 × 15 mm) on the left maxillary gingiva, with discontinuous peripheral pigmentation (yellow arrow).

**Figure 2:**
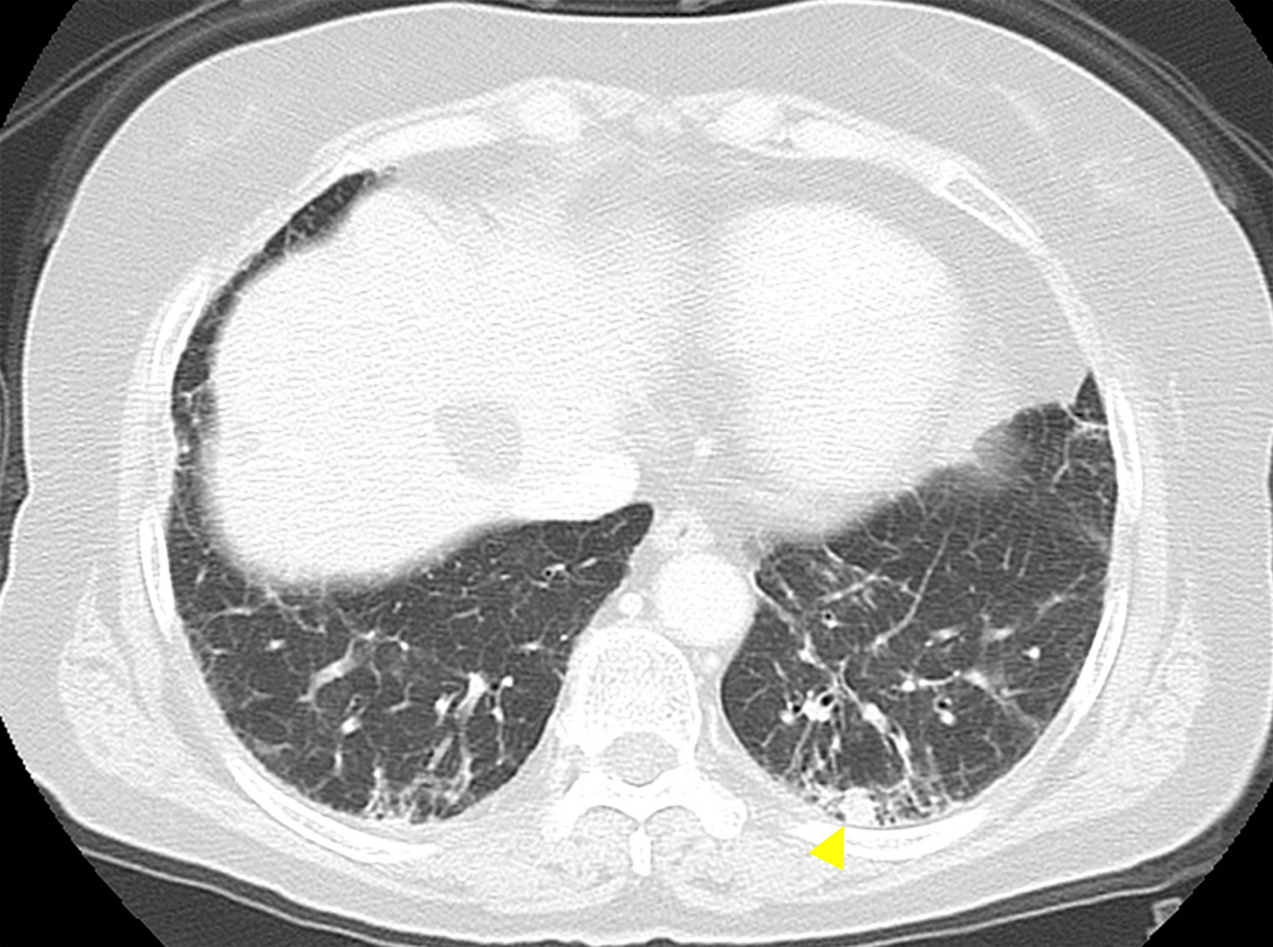
Contrast-enhanced chest CT scan showing small bilateral pulmonary nodules, suspicious for metastasis (yellow arrow).

One month later, the lesion had enlarged, and its color had changed to black (Figure 1), prompting imaging followed by an incisional biopsy. Preoperative imaging, including a chest X-ray and contrast-enhanced computed tomography (CT), revealed small peripheral pulmonary nodules in the left lower lobe and an enlarged para-aortic lymph node (Figure 2). The pulmonology consultation determined that the nodules were inaccessible for bronchoscopic biopsy, making it impossible to rule out malignancy. As a result, follow-up imaging was recommended. An incisional biopsy was performed, and histopathological analysis revealed OMM in situ.

**Figure 3:**
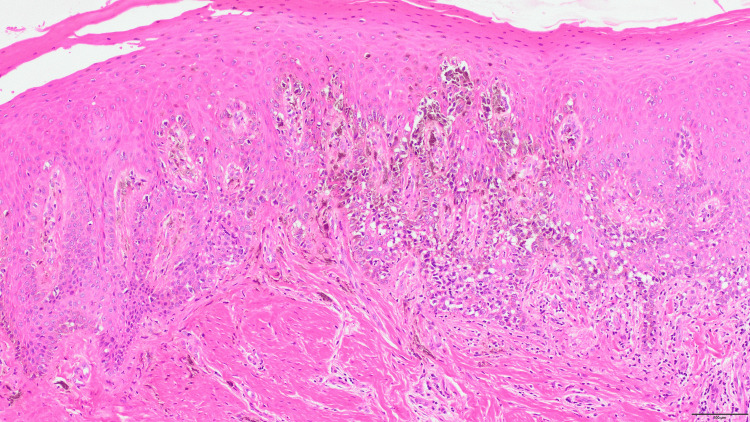
Histopathological findings of oral malignant melanoma. Hematoxylin and eosin (H&E) stain showing atypical melanocytes with melanin pigmentation in the submucosa without bony invasion.

In February 2023, a partial maxillectomy was performed under general anesthesia for a clinical diagnosis of OMM located on the left maxillary gingiva (cT3N0M0). The procedure aimed to achieve a 2-cm surgical margin around the lesion. To ensure this margin, adjacent teeth (the upper left maxillary central incisor to second maxillary molar), as well as the bony floor of the left nasal cavity and maxillary sinus, were surgically removed. Histopathological analysis of the surgical specimen confirmed OMM (Figure 3), with minimal submucosal invasion and no involvement of the bone. Surgical margins were negative.

**Figure 4:**
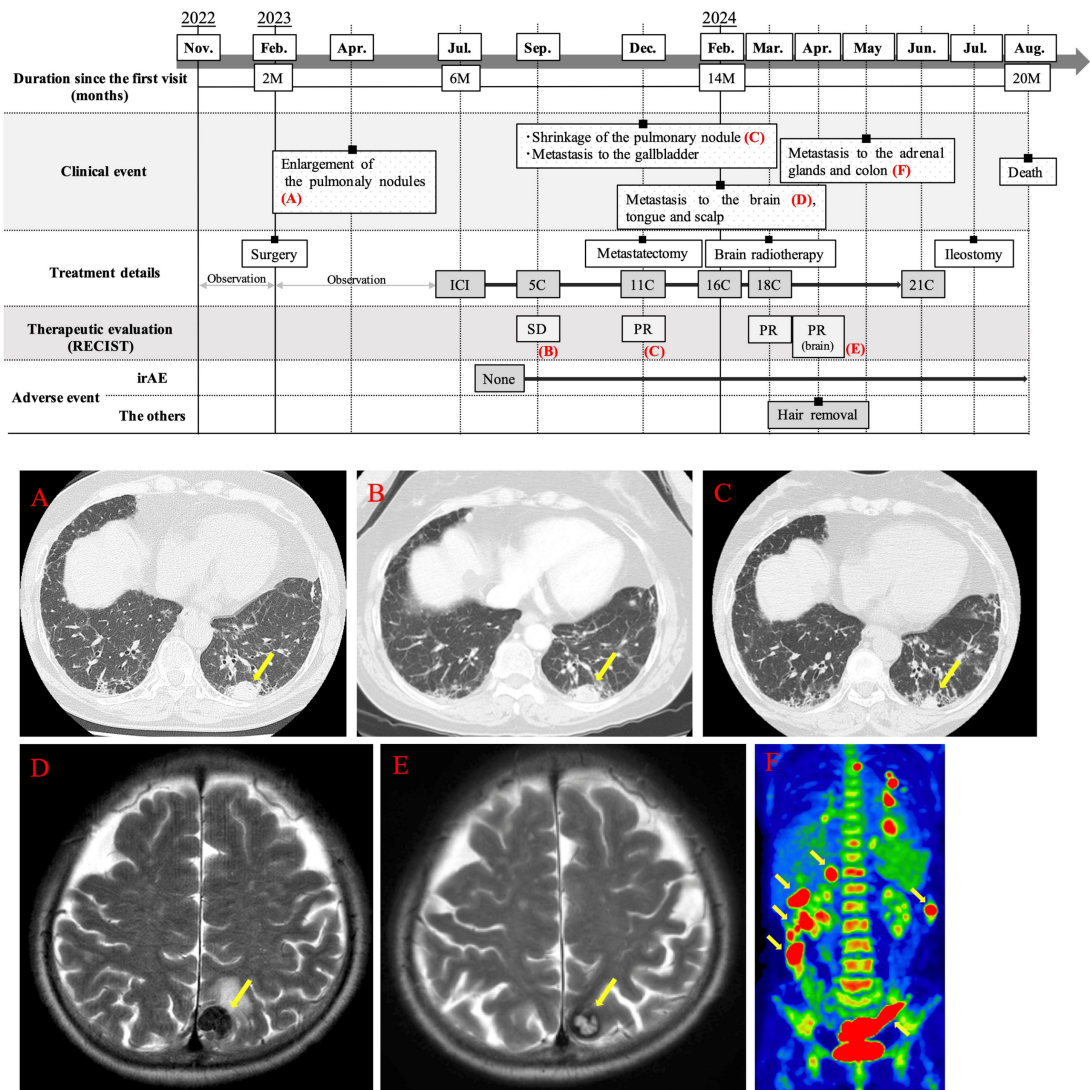
Summary of the clinical events, treatment course, including the number of nivolumab cycles, and treatment efficacy A: Chest CT scan showing an enlarged pulmonary nodule in the left lower lobe prior to immune checkpoint inhibitor therapy (yellow arrow). B: Contrast-enhanced chest CT after five cycles of immune checkpoint inhibitor therapy, demonstrating no significant change in the size of the pulmonary nodule in the left lower lobe, indicating a SD according to the RECIST (yellow arrow). C: Contrast-enhanced chest CT after 11 cycles of immune checkpoint inhibitor therapy demonstrating shrinkage of the pulmonary nodule in the left lower lobe (yellow arrow). D: Axial T2-weighted contrast-enhanced brain MRI showing shrinkage and central necrosis of the brain metastases, indicating a PR according to the RECIST (yellow arrow). E: Axial T2-weighted contrast-enhanced brain MRI showing multiple ring-enhancing metastatic lesions (yellow arrow). F: Fluorodeoxyglucose positron emission tomography showing new metastases in the adrenal glands and colon (yellow arrow).

Postoperatively, the patient was monitored on an outpatient basis. In April 2023, it was observed that the left lower lobe pulmonary nodule had enlarged (Figure 4A), and a new 5-mm black macule appeared at the posterior margin of the maxillectomy site, indicating a possible recurrence. Positron emission tomography (PET)-CT scan performed in May 2023 revealed increased uptake in the solid nodule in the left lower lobe (SUV max: 9.03, compared to 4.58 previously) and in the para-aortic lymph node (SUV max: 9.97, compared to 5.61 previously), with no abnormal uptake noted elsewhere.

In June 2023, a thoracoscopic biopsy was performed, and histopathological analysis confirmed the presence of pulmonary metastasis from OMM. BRAF V600E mutation testing yielded negative results. The PD-L1 expression, measured by the combined positive score, was ≥1%, leading to the initiation of nivolumab monotherapy. After 5 cycles of treatment, the disease remained stable, classified as stable disease according to the Response Evaluation Criteria in Solid Tumors (RECIST) (ver 1.1), with no changes in the size of the pulmonary nodules (Figure 4B). After 11 cycles, a contrast-enhanced CT revealed shrinkage of both the pulmonary nodule (Figure 4C) and the para-aortic lymph node, indicating a partial response(PR) according to the RECIST. However, a new lesion was detected in the gallbladder.

In December 2023, the patient underwent a cholecystectomy for both diagnostic and therapeutic purposes. Pathological examination confirmed the presence of metastasis of OMM to the gallbladder. In February 2024, after 16 cycles of nivolumab treatment, multiple brain metastases were detected through Magnetic Resonance Imaging (MRI) (Figure 4D). Around the same period, metastases to the tongue and the right frontal scalp also appeared.

At this point, best supportive care was proposed, but the patient was eager to continue with nivolumab. In March 2024, after completing 18 cycles of treatment, the patient was administered palliative whole-brain radiotherapy (total 30 Gy). After the radiotherapy, nivolumab was continued with the expectation of achieving a synergic effect. In April 2024, a follow-up MRI showed shrinkage and central necrosis of the brain metastases, indicating a PR according to the RECIST (Figure 4E). However, in May 2024, new metastases were detected in the adrenal glands and colon (Figure 4F). The following month, the patient complained of difficulties with defecation, and a lower gastrointestinal endoscopy revealed metastasis of OMM throughout the colon. After obtaining adequate informed consent, nivolumab was discontinued following the 21st cycle, and the patient transitioned to palliative care. During the administration of nivolumab in the present case, thorough evaluations of the patient's status and blood tests were conducted every 2 to 4 weeks. Notably, no adverse events classified as immune-related adverse events (irAEs) occurred. Additionally, there were no significant fluctuations in blood test results associated with irAEs. In July 2024, as part of palliative care, an ileostomy was performed. The patient died in August 2024, 20 months after the initial diagnosis, due to disease progression.

## Discussion

OMM typically presents as a pigmented macule, patch, or nodular lesion, most commonly affecting the palate or maxillary gingiva, and may exhibit progressive enlargement or color change over time [1]. In the present case, the lesion initially appeared as a small brown macule of uncertain nature, making differentiation from a pigmented nevus or oral melanosis challenging. It gradually enlarged and turned black, prompting us to perform an incisional biopsy. This clinical course was consistent with previously reported characteristics of OMM.

OMM is an aggressive tumor with a poor prognosis, with a reported median survival of only six to nine months [2-4]. In the present case, the patient survived 20 months after the initial diagnosis, exceeding previously reported survival durations. To the best of our knowledge, no cases of long-term survival have been reported with nivolumab monotherapy and whole-brain radiotherapy following surgical treatment for OMM. In this case, although OMM developed multi-organ metastases postoperatively and had a poor prognosis, we believe that the local synergistic effect of nivolumab monotherapy combined with radiotherapy played a role in suppressing disease progression. In this paper, we primarily discuss the selection of ICIs for unresectable OMM and their synergistic effect with radiotherapy as the primary focus.

According to the guidelines for drug therapy in MM, the first-line treatment for unresectable MM without a BRAF mutation is ICIs [5]. Recommended ICIs include combination therapy with nivolumab and ipilimumab, or monotherapy with either nivolumab or pembrolizumab. In cases of cutaneous MM or mucosal MM, adding ipilimumab to nivolumab monotherapy is expected to have an additive effect [11,12]. However, for OMM, subgroup analyses that compare combination therapy with monotherapy have not yet been conducted, and there is currently insufficient evidence to support an additive effect from combining ipilimumab with nivolumab. Ipilimumab is associated with a high incidence of Grade 3 or higher immune-related adverse events (irAEs), occurring in approximately 40% of patients [11]. There have also been reports of drug-related deaths linked to its use [12]. Therefore, careful consideration must be given when selecting this medication for OMM. In the present case, due to the insufficient evidence demonstrating ipilimumab's superiority over nivolumab and the potential risks of severe irAEs associated with ipilimumab, we chose nivolumab monotherapy as ICIs. Indeed, in the present case, nivolumab monotherapy may provide significant clinical benefits while decreasing the risk of irAEs.

However, not all metastatic lesions responded to nivolumab treatment. After completing 11 cycles of nivolumab, the patient achieved a PR concerning the lung metastases. On the other hand, subsequent metastases developed in the gallbladder, brain, and colon, indicating the emergence of tumor components that were resistant to nivolumab.

In the present case, although no neurological symptoms related to brain metastasis were observed, there was concern that such symptoms or cerebral edema could develop soon due to the rapid progression of the tumor. As a result, palliative whole-brain radiation therapy was administered. While melanoma is generally resistant to radiation, this treatment is considered effective for alleviating symptoms associated with tumor progression [13]. Additionally, there is potential for a synergistic effect when combined with ICIs. Previous reports indicate that radiation therapy can increase PD-L1 expression in melanoma cells, leading to this synergistic effect [14]. Furthermore, radiation therapy promotes tumor cell death. It releases tumor debris and antigens, which can induce adaptive T cell-mediated immune responses through mechanisms such as dendritic cell activation and cross-presentation of tumor-associated antigens. These mechanisms may ultimately contribute to suppressing tumor progression [15,16]. In the present case, whole-brain radiation therapy proved effective, resulting in a PR. Although the precise mechanism remains unclear, we believe that the local effect was achieved through the synergistic action of radiation and ICIs, as described above.

In addition to the local synergistic effects of radiation therapy and ICIs, there is a phenomenon known as the abscopal effect. This effect refers to the rare occurrence of tumor regression at a secondary site after radiation therapy is administered to a separate primary tumor [17]. However, at least in the present case, the clinical outcome following radiation therapy involved metastasis to the colon, suggesting that the abscopal effect was minimal. While the generalizability of our findings is limited due to the rarity of this disease and the fact that it is based on a single case report, our research adds to the increasing evidence supporting the use of ICIs and radiotherapy in the treatment of OMM. To establish optimal treatment strategies for this rare cancer, further case accumulation and clinical studies are required.

## Conclusions

In the present case, after surgical treatment for OMM, we treated the patient with nivolumab monotherapy followed by radiation therapy for multi-organ metastases, resulting in a survival period of 20 months. Despite the high level of malignancy, we believe that this comparatively long survival, relative to previous reports, was due not only to the effectiveness of ICIs (nivolumab monotherapy) but also to the local synergistic effect achieved by combining ICIs with radiation therapy.
